# Nutritional Status of Adolescent Afghan Refugees Living in Peshawar, Pakistan

**DOI:** 10.3390/nu13093072

**Published:** 2021-08-31

**Authors:** Anum Saeedullah, Muhammad Shabir Khan, Simon C. Andrews, Khalid Iqbal, Zia Ul-Haq, Syed Abdul Qadir, Haris Khan, Ishawu Iddrisu, Muhammad Shahzad

**Affiliations:** 1 Kabir Medical College, Department of Biochemistry, Gandhara University, Canal Road University Town, Peshawar 25000, Pakistan; anumsaeedullah1990@gmail.com; 2 Institute of Basic Medical Sciences, Khyber Medical University, Peshawar 25120, Pakistan; mshabbir.kmu@gmail.com (M.S.K.); khalid.ibms@kmu.edu.pk (K.I.); drzia@kmu.edu.pk (Z.U.-H.); qadirmasoomi@gmail.com (S.A.Q.); microbiologist53@gmail.com (H.K.); 3 School of Biological Sciences, Health and Life Sciences Building, University of Reading, Reading RG6 6AX, UK; I.Iddrisu@pgr.reading.ac.uk

**Keywords:** malnutrition, vulnerable, stunting, micronutrients deficiency, anemia, vitamins

## Abstract

Pakistan has hosted millions of Afghan refugees over the last several decades. Due to poor socioeconomic status, food insecurity and inadequate access to health care, these refugees are considered to be at high risk of malnutrition. Previous studies on nutritional assessment of high-risk populations (refugees) have focused mainly on women and children (0–59 months). The current study aims to assess nutritional status of adolescent Afghan refugees; the population who are equally vulnerable to malnutrition and its consequences. In this cross sectional study, the nutritional status of 206 adolescent (10–19 years old) Afghans boys and girls living in a refugee camp in Peshawar, Pakistan was assessed using standard methods. The results indicate a prevalence of stunting, thinness, and overweight and obesity at 35.3%, 4.4% and 14.8%, respectively. Furthermore, there was a significantly high prevalence of micronutrient deficiencies (vitamin D, 80.5%; vitamin B12, 41.9%; and folate, 28.2%); and anemia (10.1%). Together, these findings indicate that this vulnerable population group suffers from the double burden of malnutrition and are thus at serious risk of impaired psychosocial cognitive development, general ill-health and diminished wellbeing. This study therefore highlights the urgent need to include adolescents in regular screening and intervention programs of such at-risk populations.

## 1. Introduction

Pakistan, with an estimated population of >208 million people, is the 6th most populous country of the world [1]. Since its independence in 1947, the country has faced an increase in serious issues related to its security, economy and the health of its population [2]. The World Bank categorizes Pakistan among the low and middle income countries [3] with an annual household income per capita at just $587 [4]. Despite its widely prevalent financial and political instability, the country ranks third in the word for the number of refugees hosted, according to the latest reports from the United Nations High Commission for Refugees (UNHCR) (2020) [5]. The majority of these refugees are Afghans who have fled their country and took shelter in Pakistan during the 1978–1979 Soviet and 2001 U.S./NATO/ISAF-led occupation of Afghanistan. However, most have now returned to their homeland as part of UNHCR volunteer repatriation drive. Nevertheless, an estimated 1.4 million registered [5] and further 1 million unregistered Afghan refugees still remain in Pakistan of which more than 60% reside in Khyber Pakhtunkhwa province.

Refugees constitute a vulnerable population group who usually undergo a number of traumatic events during their journey from their home country and during settlement within their new host country. As a result, refugees are at high risk of nutrition and health disparities [6], which includes increased susceptibility to specific communicable and non-communicable diseases [7,8,9]. Afghan refugees in Pakistan are a unique vulnerable population who suffer food insecurity and malnutrition in their both their home and host countries. Food insecurity and malnutrition, especially micronutrient deficiency in children, are a common public health issue in both counties. This is evident from the recent National Nutrition Surveys reporting a staggering prevalence of malnutrition in both Pakistan and Afghanistan [10,11]. Adolescent population is no exception. The 2018 national nutrition survey of Pakistan reported that 21.1% of adolescent boys and 11.8% of adolescent girls are underweight. Overweigh (boys = 10.2%; girls = 1.4%) and obesity (boys = 7.7%; girls = 5.5%) is also common. More than half of the adolescent girls (56.6%) were anemic although, severe anemia was only found in 0.9%. Malnutrition can have serious consequences upon child development, morbidity and mortality [12]. Till now, the majority of studies assessing nutritional status in emergency-affected populations (refugees and displaced populations) and resource-poor countries such as Pakistan have focused mainly on women and children (6–59 months of age). In most of these studies, adolescents were not considered at high risk of nutritional deficiency and hence were not assessed clinically.

Adolescence is the transitional stage of life between childhood and adulthood. According to World Health Organization (WHO), adolescents constitute those between the age of 10–19 [13]. Adolescence represents the second growth spurt period after infancy characterized by rapid growth in stature, muscles and body fats. In order to meet the growing demands of physical growth, reproductive maturation and cognitive development, nutritional requirements (both macro and micronutrients) are as high or higher than any other age groups [14,15,16]. For example, the daily energy requirements of adolescents (2420 kcal) is highest than any other age group. Similarly, adolescents also require higher daily amount of micronutrients and calcium than adults and children [17].

Malnutrition (both micro and macronutrient deficiency) is common in refugee camps and displaced populations primarily due to food insecurity, poor socio-economic conditions, inadequate food intake and disease burden [18,19,20]. Wasting, stunting and multiple micronutrient deficiencies (such as iron, vitamin A) among adolescent refugee populations of Kenya and Nepal and have been reported previously [21,22]. Recent studies from refugee camps in Somalia and Turkey have also reported high prevalence of thinness and stunting among adolescents [17,23] resulting in significant negative impacts on health and wellbeing [24]. Moreover, adolescents generally have little contact with health or nutrition services and in the absence of specific nutrition intervention programs targeting adolescents, there is increased risk of malnutrition and subsequent deleterious health consequences in this vulnerable group.

The current study was undertaken to assess the prevalence of malnutrition in adolescent Afghan refugees. The aim was to provide data that would indicate the degree and extent of malnutrition within this vulnerable population group. As such, this survey represents as an important step towards determining the need for intervention strategies directed at breaking the cycle of intergenerational malnutrition and poverty, and the health-related consequences.

## 2. Materials and Methods

### 2.1. Study Setting

The study was conducted in the Khazana Refugee Camp. The camp is located in Peshawar, the capital city of Khyber Pakhtunkhwa province of Pakistan. It is among the oldest refugee camp established back in 1979. Currently, the camp is home to 900 refugee families with a total population of around 5000. The camp is administratively controlled by government of Pakistan through Commissionerate Afghan Refugees Khyber Pakhtunkhwa (KPCAR).

### 2.2. Study Design and Participant Recruitment

A randomized, community based, cross-sectional study design was employed among 206 adolescent boys and girls residing in Khazana refugee camp, Peshawar from March–April 2020. The sample size was calculated using OpenEpi Epidemiologic Statistics software (www.OpenEpi.com; Version updated 6 April 2013, accessed on 11 January 2020). A simple, random sampling technique was utilized to obtain the required number of participants from among the 921 refugee families. Eligible participants were adolescents, aged 10–19, who were apparently healthy and not using antibiotic medication, nutritional supplements, prebiotics or probiotics (in food products or as supplements), laxatives, anti-spasmodic or anti-diarrhea drugs (e.g., Orlistat, Lactulose, etc) during the study period or in the past two months. Physically handicapped, pregnant and lactating adolescent girls, non-consenting individuals and those lacking the mental status to give informed consent were excluded from the study. We also excluded adolescents living outside of the specific study locality.

Ethics approval of the study was obtained from Research Ethics Committee of Khyber Medical University and administrative approval from Commissionerate Afghan Refugees, Peshawar. Before the start of the study, 3 information sessions were held with community elders and local administration of the refugee village in order to facilitate recruitment and data collection especially from female participants. Only female data collectors were allowed to visit the homes of female participants, and collect data and samples. The participant information sheet was translated into the local language (Pashto and Dari) and was provided to participants (age 18–19 years), and the parents and legal guardians of those age <18 years. The detailed data collection procedure was also explained verbally and any queries were answered before parental permission and written informed consent were obtained.

### 2.3. Collection of Socio-Demographic Data

A pre-designed, interviewer administered, structured questionnaire containing both open and close ended questions was used to collect socio-demographic information of the participants such as name, age, gender, ethnicity, education, occupation and household income.

### 2.4. Anthropometric Measurements

Height and weight of the participants were recorded following standard methods. Before height measurement, the participants were asked to remove footwear and socks and stand stable on a wall mounted Stadio meter (Seca, UK) in the Frankfurt horizontal plane position. Height was recorded three times, to the nearest 0.1 cm, and the average measurement was considered the final weigh for each participant. Participant’s weight was recorded in kilograms to the nearest 0.1 kg using a calibrated electronic scale (Seca, UK). Body mass index (BMI) was computed as the fraction of weight to the squared height (kg/m^2^). All the anthropometric data were collected by two researchers fully trained by the principal investigator.

### 2.5. Biochemical Analysis

Non-fasting whole-blood samples from the antecubital vein were collected by a trained phlebotomist using butterfly needles and plastic vacutainers (BD Diagnostics, Basel, Switzerland). At least 5 mL of blood were drawn into pre-chilled tubes containing Ethylenediaminetetraacetic acid (EDTA). Whole blood was used for complete blood count, hemoglobin, hematocrit and mean corpuscular volume using an automated hematology analyzer (Sysmex XP-100, 19 Jalan Tukang, Singapore). Plasma was separated by centrifugation within one hour of sample collection, aliquots were prepared (200 µL) and stored at −80 °C. These samples were used to assess plasma vitamin D using a 25-OH vitamin D Diasorin radioimmunoassay ELISA kit (Euroimmun, Germany) following manufacturer’s instructions. For serum based biomarkers assessment, 5 mL of blood were collected in collection tubes containing silica clot activator. The samples were left on ice for 30 min followed by centrifugation to separate serum and storage of aliquots (200 µL) at −80 °C. Serum samples were used to assess ferritin, vitamin B12 and folate using the Abbott Architect i2000 analyzer (Abbott Diagnostics, Zug, Switzerland).

### 2.6. Operational Definitions

Anemia was defined on the basis of hemoglobin concentration following WHO recommended age and sex specific cut off points; <11.5 mg/dL (for both girls and boys age 10–11 years), <12 mg/dL (for both girls and boys age 12–14 years and non-pregnant girls age 15 and above), <13 mg/dL for boys age 15 years and above [25]. Serum ferritin < 15 µg/L indicated depleted iron stores [26]. The cut off point for vitamin B12 was 203 pg/mL [27]. Folate concentration was categorized as deficiency (<3 ng/mL), possible deficiency (3–5.9 ng/mL) and normal (6–20 ng/mL), as reported by WHO [28]. Serum 25-hydroxyvitamin D [25(OH)D] < 30 ng/mL was defined as vitamin D deficiency [29]. Thinness, overweight and obesity were defined as BMI-for-age Z score BAZ < −2, BAZ > 1 and BAZ > 2 WHO reference data [30]. Similarly, the participants were categorized as stunted when the height-for-age Z score was <−2 SD from the median value of WHO reference data [30].

### 2.7. Statistical Analysis

Age categories, education attainment of the respondents, family size, educational attainment of the female head of the household and income quartiles were described as frequency and percentages. Statistical differences in the background characteristics between the two genders was assessed using *t*-test and difference of proportions were assessed using two-proportion Z-test. Thinness and over-weight/obesity were assessed using BMI-for-age Z-score and stunting was assessed using height-for-age Z-score. Observations with extreme BMI-for-age Z-score values above 5 Standard Deviation (SD) and below −5 SD were removed from the BMI-for-age analysis. Similarly, observations with extreme height-for-age Z-score values above 6 Standard Deviation (SD) and below −6 SD were removed from the height-for-age Z-score analysis. Statistical difference between nutritional status of boys and girls for different categories was assessed using two-proportion test. BAZ and HAZ were assessed using WHO-anthro plus version 1.0.4. All other analysis were done using SAS Enterprise Guide Version 7.1.

## 3. Results

### 3.1. Socio-Demographic Characteristics of the Participants

A total of 206 adolescents (103 boys and 103 girls) were included in thestudy. Participants with missing information (*n* = 1) on weight, height, or age were excluded from the anthropometric analysis but were retained for the estimation of micronutrient prevalence. Demographic and socio-economic characteristics of the study participants are shown in Table 1. The mean age of the participants was 13.4 ± 2.9 years. There was no statistical difference between the mean ages of boys and girls adolescents in the study. Two-thirds of the participants were 10–14 years. The majority (61%) of the participants had primary-level education with no statistical difference between boys and girls. Half (49.5%) of the participants had 10–19 family members. Three-fourths of the girls household heads had no formal education.

### 3.2. Anthropometric Measurements

The average height and weight of the participants were 141.5 ± 18.3 cm and 38.2 ± 12.3 kg, respectively. The overall prevalence of thinness, stunting, overweight and obesity among adolescent boys and girls refugees is given in Table 2. BMI-for-age classification showed around 4.4% of the participants were thin with a higher proportion (8%) of boys being thin as compared to girls participants (*p* = 0.02). No major differences in thinness were observed between the two age groups i.e., 10–14 and 15–19 years. Prevalence of overweight and obesity were 11.8% and 3%, respectively, among the study subjects. Compared to girls, overweight and obesity were two times more prevalent in boys. Overall stunting among the study subject was 35.3%. High proportion of stunting was observed among 11–14 years old as compared to 15–19 years old participants.

**Figure 1:**
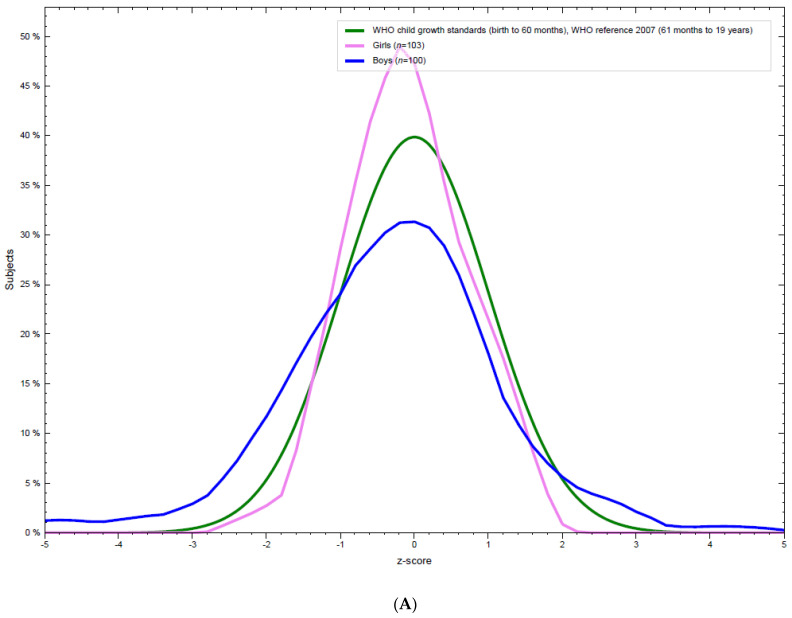

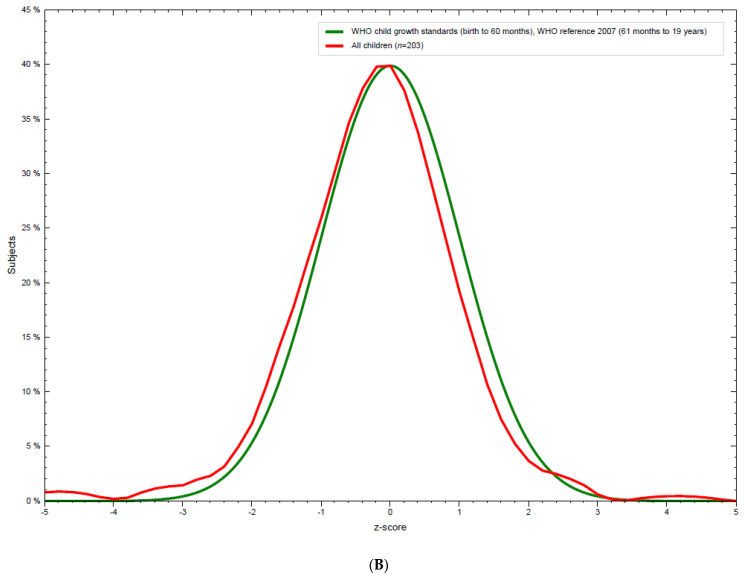
(**A**) BMI-for-age distribution of adolescent, boys and girls Afghan refugees. (**B**) Distribution of BMI-for-age of all the adolescents as compared to the WHO standard.

BMI-for-age (BFA) distribution of the study participants is shown in Figure 1A,B. Compared to WHO standards, BFA distribution for both boys and girls showed marked differences in the distribution. BFA for women had higher mean and narrower spread showing less extreme BFA as compared to the expected growth standards. Whereas boys had lower mean BFA with wider tails reflecting extreme BFA values.

However, combined BFA distribution for all participants does not show major deviation from the normal curve (Figure 1B).

**Figure 2:**
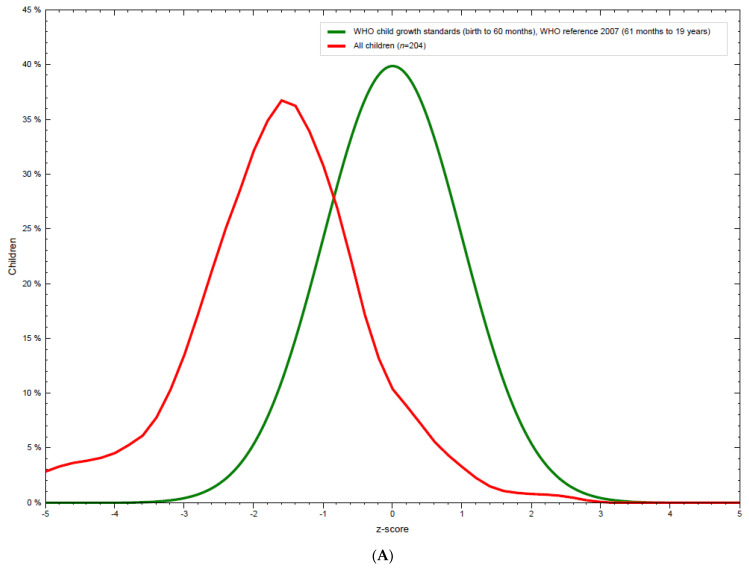

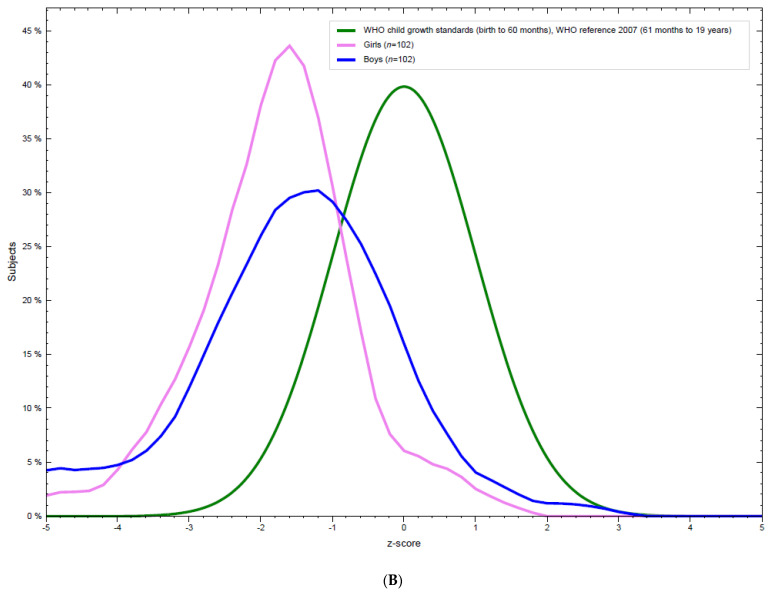
(**A**) Distribution of height-for-age of the adolescents compared to WHO standard. (**B**) Distribution of height-for-age stratified by sex of the adolescents.

One in three adolescents also showed stunted growth with highest prevalence in young adolescent age 10–14 years (Table 2). No statistically significant difference was observed between height-for-age z-score (HAZ) for boys and girls. HAZ distribution of all the participants showed significant deviation from the WHO growth standard curve (Figure 2A); the distribution of which is shifted to the left with low mean HAZ. HAZ distribution for both sexes are shown separately in Figure 2B. Both showed significant deviation from the WHO growth standard with higher stunting observed in boys as compared to girls.

### 3.3. Blood Based Biomarkers of Nutritional Status/Micronutrient Status

The mean blood hemoglobin concentration of the participants was 13.0 ± 1.4 mg/dL. Female adolescents had slightly lower plasma hemoglobin concentration (12.9 ± 1.5 mg/dL) than boys (13.2 ± 1.2 mg/dL) but the differences were not significant. Based on WHO cutoff points, anemia was present in 23 (11.2%) of the participants with no significant differences between genders (boys vs. girls) and age groups (10–14 vs. 15–19 years). This pattern was reflected in the serum ferritin concentrations indicating potential iron deficiency in 19 (9.6%) of the participants. The majority of the participants had multiple micronutrients deficiencies, the most prevalent being vitamin D deficiency present in 80.5% of the participants followed by vitamin B12 (41.9%) and folate (28.2%) deficiencies. Iron, folate and vitamin D deficiencies were significantly higher (*p* < 0.01) among girls adolescents whereas the prevalence of vitamin B12 deficiency was higher among the male adolescents. The micronutrient status of the participants is summarized in Table 3.

## 4. Discussion

The current study reports the presence of malnutrition across the nutritional spectrum among Afghan adolescents, which is one of the largest protracted refugee communities in the world. The prevalence of stunting, thinness and overweight/obesity along with multiple micronutrient deficiencies in this population is representative of the double burden trend of malnutrition (under and over-nutrition) in low and middle income countries [31]. Overall, more than half (54.5%) of the adolescent population in the refugee camp presented with at least one form of malnutrition, the commonest being stunting (35.3%) and overweight/obesity (14.8%). This study also provide evidence that malnutrition affects both male and female adolescents across the age spectrum (younger and older adolescents). This study also reports that stunting was more common in younger adolescents (10–14 years of age) and obesity was only evident in boys. Finally, the study identified anemia and a significantly high prevalence of micronutrient deficiencies for iron, folate, vitamin D and vitamin B12.

The current study was conducted in a refugee village (ARV) where the inhabitants generally have poor socioeconomic status and education background, especially for girls. As indicated in the 2020 UNHCR report, only 31% of the Afghan refugees live in refugee villages [32]; indeed, the majority are settled in rural and urban communities primarily due to the poor standard of living associated with refugee camps. As such, malnutrition in such populations is unsurprising, as is evident from the recent national nutrition survey in both Afghanistan (country of origin) [10] and Pakistan (host country) [11]. The survey reported here indicates that the prevalence of underweight, overweight and obesity in Afghan adolescent refugees is slightly lower than that nationally and provincially (Khyber Pakhtunkhwa where the ARV is located) average of prevalence in Pakistan, however, the findings indicate the same general pattern with high prevalence in male compared to female adolescents [11]. Similarly, the stunting prevalence reported here is also lower than the national (40.2%) and provincial averages (40.0%) in Pakistan. This discrepancy might be due to the age differences in the population groups considered since the NNS of Pakistan reported stunting prevalence only in children under 5 years of age, not adolescents. Previous nutritional assessment studies on vulnerable adolescents (such as refugees, street children and those with a low socioeconomic background) have also reported variable prevalence of stunting from as low 9.2% to as high as 54% [17,18,33,34]. Such variability may be due to differences in study population group settings (e.g., length of stay in refugee camps) and/or data collection processes.

We have also high prevalence of vitamin D deficiency among adolescent Afghan refugees. These findings are in concordance with other studies in refugees [18,35,36]. Deficiency was more common in girls (90.5%) than boys (70.5%). The primary reason for this raised vulnerability in the female adolescents seems is likely to be cultural. Women in Afghanistan and local Pashtun areas in Pakistan generally spend most of their time inside their homes. Even during visits to the outside, they are covered in concealing clothing and a face veil (locally known as a “shuttle cock burqa”), thus limiting sun exposure resulting in decreased vitamin D synthesis. Vitamin D deficiency in adolescents is generally asymptomatic but can have severe health-related consequences including osteomalacia and increase susceptibility to infectious and autoimmune diseases [18].

The current study also report a relatively high prevalence of vitamin B12 (41.9%) and folate deficiency (28.2%) even though only 11.2% of the adolescents were anemic. Similar findings have also been reported in Afghan refugees in Australia [37] but with a much lower magnitude than the current study. Of the many possible reasons for these vitamin deficiencies, poor socioeconomic conditions, food insecurity and high costs of vitamin B12 rich foods could be major contributors to the high prevalence of vitamin B12 deficiency [35]. Vitamin B12 deficiency is difficult to diagnose clinically as such patients mainly present with vague symptoms such as paresthesia, memory impairment, mood and personality changes and loss of appetite and weight. However, severe deficiency can have serious deleterious effects in later life such as degeneration of the spinal cord and increased risk of myocardial infarction and stroke thereby necessitating routine screening in this vulnerable population [35]. Based on the prevalence rate, anemia and iron deficiency are mild public health issues in this population group. These finding are similar to a recent study conducted in a resource poor community in the same area of Pakistan [38] but markedly different from the national nutrition survey of Pakistan [11] and other studies [39] that report anemia prevalence in >50% of the population. This was an unexpected finding as the routine dietary intake in these population groups include wheat as a staple food which is low in readily bioavailable heme iron [38]. A possible reason to this might be the culinary practices in these population that involve use of iron-based cocking pots and utensils. The near-optimum blood hemoglobin concentration reported here might thus be due to leaching of iron from the cocking pots to the food as reported by a study in Malawi [40] and highlighted by the condition of Bantu siderosis [41]. However, further research is needed to confirm this.

This study is the first to report on the nutritional status of adolescent Afghan refugees that has employed a comprehensive screening approach. The findings provide evidence of relevance to the local authorities and donor agencies for requirement for regular nutrition screening of adolescent population in the refugee camps in order to enable interventions for the promotion of health, and protection against disease and future disability. However, the study reported has several limitations. Firstly, the relatively small sample size used in the study means that the results cannot be generalized to the entire adolescent refugee population. Secondly, it is a cross sectional study conducted at a single time point in a single refugee camp and therefore the data cannot be compared with adolescent populations in other refugee camps or outside refugee camps in the urban and rural communities. Third, lack of access to other crucial information on health and healthcare access further limits the utility of this study.

## 5. Conclusions

The study findings presented here suggest that adolescent Afghan refugees are affected by the double burden associated with malnutrition. Stunting and micronutrient deficiencies are major problems while anemia, thinness and overweight/obesity are mild public health issues. There is an urgent need for further research and regular nutritional screening, health and nutrition education, and counselling services in this vulnerable population in order to more fully understand and address the problem of malnutrition with this vulnerable population group.

## Figures and Tables

**Table 1:** Demographic and socio-economic characteristics of the study participants *.

Characteristics	Total	Boys	Girls	*p*-Value
Age **	13.4(2.9)	13.1 (2.8)	13.(3.1)	0.4
Age Cat				
10–14	134 (65)	74 (71.8)	60 (58.3)	0.04
15–19	72 (35)	29 (28.2)	43 (41.7)	0.04
Education				
No formal Education	40 (19.5)	9 (8.8)	31 (30.1)	<0.005
Primary Level	125 (61)	68 (66.7)	57 (55.3)	0.1
High school level	37 (18)	22 (21.6)	15 (14.6)	0.19
College & University	3 (1.5)	3 (2.9)	0 (0)	0.15
Family size				
1–4	6 (2.9)	5 (4.9)	1 (1)	0.09
5–9	77 (37.4)	42 (40.8)	35 (34)	0.30
10–19	102 (49.5)	47 (45.6)	55 (53.4)	0.25
20 or more	20 (9.7)	8 (7.8)	12 (11.7)	0.34
Missing	1 (0.5)	1 (1)	0 (0)	0.31
Members younger than 14 years	4.0	4.0	4.4	0.27
Educational Attainment of Female (head/spouse)				
No formal Education	159 (77.6)	83 (81.4)	76 (73.8)	0.10
Primary Level	19 (9.3)	12 (11.8)	7 (6.8)	0.11
High school level	24 (11.7)	7 (6.9)	17 (16.5)	0.03
College & University	3 (1.5)	0 (0)	3 (2.9)	0.08
Income quartiles				
Q1	52 (25.4)	22 (21.6)	30 (29.1)	0.25
Q2	55 (26.8)	26 (25.5)	29 (28.2)	0.66
Q3	43 (21)	24 (23.5)	19 (18.4)	0.37
Q4	55 (26.8)	30 (29.4)	25 (24.3)	0.41

* Given are counts (percentages) except for the variable age. ** Age is expressed as mean (standard deviation).

**Table 2:** Prevalence of thinness, overweight/obesity and stunting in afghan refugee adolescents.

Characteristics	Total	Boys	Girls	*p*-Value
BMI-for-age Z-score (10–14)				
**Thinness (% < −2 SD)**	6 (4.6)	6 (8.5)	0 (0)	<0.01
**Normal (% > −2SD & ≤1SD)**	177 (80.10	78 (69)	99 (93.3)	<0.01
**Over-weight (% > 1 SD)**	15 (11.5)	11 (15.5)	4 (6.7)	0.05
**Obesity (% > 2 SD)**	5 (3.8)	5 (7.0)	0 (0)	0.01
BMI-for-age Z-score (15–19)				
**Thinness (% < −2 SD)**	3 (4.3)	2 (6.9)	1 (2.3)	0.12
**Over-weight (% > 1 SD)**	9 (12.5)	2 (6.9)	7 (16.3)	0.04
**Obesity (% > 2 SD)**	1 (1.4)	1 (3.4)	0 (0)	0.06
Stunting (% < −2 SD)				
**10–14**	52 (39.1)	27 (37.0)	25 (41.7)	0.48
**15–19**	20 (28.2)	8 (27.6)	12 (28.6)	0.87

**Table 3:** The micronutrient status of the participants.

Characteristics	Total	Boys	Girls	*p*-Value *
**All age groups**				
Hemoglobin (mg/dL) (Mean ± SD)	13.0 ± 1.4	13.2 ± 1.2	12.9 ± 1.5	
Presence of anemia (based on WHO Hb cut-off points)				
**Non-anemic**	170 (89.9)	76 (88.4)	94 (91.3)	0.51
**Anemic**	19 (10.1)	10 (11.6)	9 (8.7)	0.54
Serum Ferritin (ng/mL) (Mean ± SD)	48.7 ± 41.0	51.6 ± 45.5	45.7 ± 35.9	
Iron status (based on WHO ferritin cutoffs)				
Normal	178 (90.4)	94 (94.9)	84 (85.7)	0.03
Deficient	19 (9.6)	5 (5.1)	14 (14.3)	0.03
Folate (ng/mL) (Mean ± SD)	5.2 ± 2.4	4.2 ± 2.0	5.7 ± 2.6	
Folate status on the basis of WHO cutoffs				
Normal	32 (15.5)	26 (25.2)	6 (5.8)	<0.01
Possible deficiency	116 (56.3)	58 (56.3)	58 (56.3)	0.99
Deficiency	58 (28.2)	19 (18.4)	39 (37.9)	<0.01
Vitamin B12 (pg/mL) (Mean ± SD)	275.3 ± 170	241.5± 138.6	308.7 ± 191.2	
Vitamin B-12 status on the basis of WHO cutoffs				
Normal	111 (58.1)	45 (47.4)	66 (68.8)	<0.01
Deficient	80 (41.9)	50 (52.6)	30 (31.3)	<0.01
Vitamin-D (ng/mL) (Mean ± SD)	22.4 ± 8.0	26.7 ± 6.3	18.0 ± 7.1	
Vitamin D deficiency				
Normal	37 (19.5)	28 (29.5)	9 (9.5)	<0.01
Deficient	153 (80.5)	67 (70.5)	86 (90.5)	<0.01
Iron (µg/dL)	81.2 ± 31.4	83.2 ± 31.8	79.3 ± 31	
**Age group (10–14)**				
Presence of anemia (based on WHO Hb cut-off points)				
Non-anemic	112 (92.6)	55 (90.2)	57 (95)	0.31
Anemic	9 (7.4)	6 (9.8)	3 (5)	0.31
Iron status (based on WHO ferritin cutoffs)				
Normal	120 (93)	68 (95.8)	52 (89.7)	0.17
Deficient	9 (7)	3 (4.2)	6 (10.3)	0.17
Folate status on the basis of WHO cutoffs				
Normal	16 (11.9)	13 (17.6)	3 (5)	0.03
Possible deficiency	81 (60.4)	44 (59.5)	37 (61.7)	0.79
Deficiency	37 (27.6)	17 (23)	20 (33.3)	0.18
Vitamin B-12 status on the basis of WHO cutoffs				
Normal	68 (55.7)	31 (46.3)	37 (67.3)	0.02
Deficient	54 (44.3)	36 (53.7)	18 (32.7)	0.02
Vitamin D deficiency				
Normal	27 (22.1)	21 (31.3)	6 (10.9)	0.01
Deficient	95 (77.9)	46 (68.7)	49 (89.1)	0.01
**Age group (15–19)**				
Anemia prevalence (based on WHO Hb cut-offs)				
Non-anemic	58 (85.3)	21 (84)	37 (86)	0.82
Anemic	10 (14.7)	4 (16)	6 (14)	0.82
Iron status (based on WHO ferritin cutoffs)				
Normal	58 (85.3)	26 (92.9)	32 (80)	0.14
Deficient	10 (14.7)	2 (7.1)	8 (20)	0.14
Folate status on the basis of WHO cutoffs				
Normal	16 (22.2)	13 (44.8)	3 (7)	<0.01
Possible deficiency	35 (48.6)	14 (48.3)	21 (48.8)	0.99
Deficiency	21 (29.2)	2 (6.9)	19 (44.2)	<0.01
Vitamin B-12 status on the basis of WHO cutoffs				
Normal	43 (62.3)	14 (50)	29 (70.7)	0.10
Deficient	26 (37.7)	14 (50)	12 (29.3)	0.10
Vitamin D deficiency				
Normal	10 (14.7)	7 (25)	3 (7.5)	0.04
Deficient	58 (85.3)	21 (75)	37 (92.5)	0.16

* *p*-value of two-proportion tests.

## Data Availability

Not applicable.
